# A Path Analysis of Adolescent Athletes’ Perceived Stress Reactivity, Competition Appraisals, Emotions, Coping, and Performance Satisfaction

**DOI:** 10.3389/fpsyg.2019.01151

**Published:** 2019-05-15

**Authors:** Darren M. Britton, Emma J. Kavanagh, Remco C. J. Polman

**Affiliations:** ^1^School of Sport, Health, and Social Sciences, Solent University, Southampton, United Kingdom; ^2^Department of Sport & Physical Activity, Bournemouth University, Bournemouth, United Kingdom; ^3^School of Exercise and Nutrition Sciences, Queensland University of Technology, Brisbane, QLD, Australia

**Keywords:** stress reactivity, stress appraisal, emotion, coping, adolescence, performance

## Abstract

This study examined a path analysis of adolescent athletes’ individual differences in perceived stress reactivity, competition appraisals, emotions, coping, and performance satisfaction. The study aimed to extend an analysis by Nicholls et al. (2012) and further validate the use of the Perceived Stress Reactivity Scale for Adolescent Athletes (PSRS-AA). Adolescent athletes (*N* = 229, *M* age = 18.55, *SD* = 2.40) completed the PSRS-AA followed by a measure of competition appraisals less than 1 h before a competitive event. Within an hour after the competitive event, participants completed a retrospective assessment of emotions, coping strategies, and subjective performance. A path analysis revealed that perceived stress reactivity had direct and indirect effects on the appraisal of higher stressor intensity, lower perceived control, higher perceived threat, negative emotions, and maladaptive coping. Increased threat, positive and negative emotions, and maladaptive coping were associated with performance satisfaction. However, task-orientated coping was not associated with performance satisfaction. The present study enhances and refines the validity of the PSRS-AA for assessing adolescent athletes’ perceived stress reactivity. Further strengths and weaknesses of the present study are discussed, along with recommendations for practitioners aiming to support adolescent athletes with high levels of stress reactivity.

## Introduction

Stress is an ongoing transaction between an individual and their environment (Lazarus and Folkman, 1987). Environmental demands encountered by individuals are commonly referred to as “stressors” (Lazarus and Folkman, 1987; Fletcher et al., 2012). Stressors, depending upon how they are appraised, can produce numerous negative physical, psychological, and behavioral responses from an individual that can significantly affect athletic performance and satisfaction, particularly if individuals do not cope with them adaptively (Lazarus and Folkman, 1987; Lazarus, 2000; Fletcher et al., 2006; Nicholls et al., 2012; Laborde et al., 2016; Arnold et al., 2017). Specifically, based upon a path analysis performed by Nicholls et al. (2012), appraisals of challenge prior to sporting competition are associated with positive emotions, task orientated coping strategies, and increased performance satisfaction, while threat appraisals are associated with negative emotions, distraction and disengagement orientated coping strategies and decreased performance satisfaction.

Competitive sport can produce multiple stressors which young athletes must contend with (Nicholls et al., 2005; Reeves et al., 2009). These include stressors related directly and indirectly to their sports performance (Neil et al., 2011), and those associated with the physical, emotional, and social developments of adolescence (Compas et al., 2001; van Rens et al., 2016). An inability to cope with stressors has been cited as one of the main causes of both burnout and dropout in youth sport (Goodger et al., 2007; Crane and Temple, 2015), and one of the reasons why some talented youth athletes fail to achieve success (Holt and Dunn, 2004). Therefore, assisting young athletes in coping more adaptively with the stressors they experience during this challenging period is important not just for enhancing performance in active individuals, but also maintaining levels of participation and protecting health.

Adolescence is an important period where an individual’s stress reactivity (SR), plus their repertoire of coping strategies, develops (Blakemore and Choudhury, 2006; Nicholls et al., 2009; Romeo, 2010). SR has been defined as an individual difference reflecting the broad variability in responses to stressors (Boyce and Ellis, 2005; Ellis et al., 2005; Schlotz et al., 2011a,b; Schlotz, 2013). Hyper-reactivity in adolescents has been associated with internalizing symptoms (negative emotionality, anxiety, and depression; Granger et al., 1994; Allwood et al., 2011; Marceau et al., 2012; Lopez-Duran et al., 2015).

Given the large number of stressors adolescent athletes face (Nicholls et al., 2005; Reeves et al., 2009), one’s level of SR would be a significant risk factor to youth athletes, increasing or decreasing the likelihood of experiencing more adverse reactions to environmental demands. More intense stress reactions in response to these demands would likely be harder to cope with, thus increasing the potential for burnout and dropout from sport. Furthermore, exposure to a large number of stressors during adolescence (sport-related or otherwise) is also likely to affect and influence one’s level of SR (Romeo, 2010).

The development of SR is highly dependent on environmental influences during childhood and adolescence, particularly exposure to both adversity and support (Boyce and Ellis, 2005; Romeo, 2010). A recent meta-analysis has revealed how an increased exposure to adverse childhood experiences influences the development of maladaptive reactivity to stress (Hughes et al., 2017). However, exposure to a moderate level of adverse experiences among athletic populations have been associated with adaptive physiological responses under pressure (Moore et al., 2018).

**FIGURE 1:**
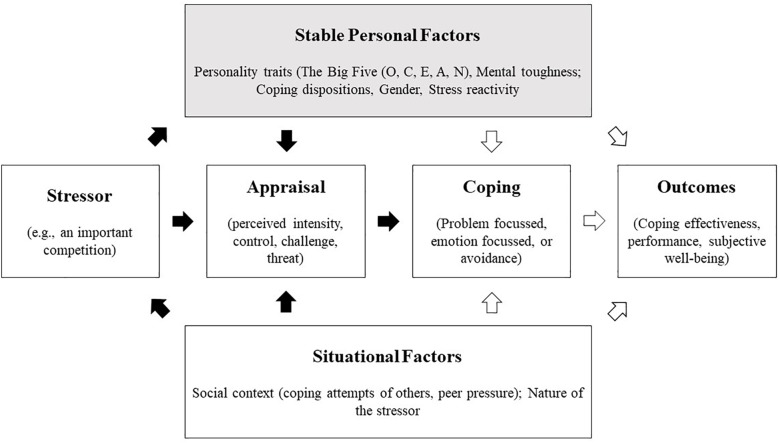
Conceptual framework illustrating how stable and situational factors directly and indirectly influence the stress and coping response (Kerdijk et al., 2016). Black arrows represent direct effects, while white arrows indicate indirect effects. (Adapted with permission granted from corresponding author R. Polman).

Numerous individual differences and personal factors have been associated with the way in which athletes appraise and cope with stressors (see Figure 1; Kerdijk et al., 2016), including gender (Kaiseler et al., 2012b) the Big Five personality traits (Kaiseler et al., 2012a), mental toughness, (Kaiseler et al., 2009), and maturity (Nicholls et al., 2009, 2013, 2015). However, less research has focussed upon individual differences in adolescent athletes’ SR.

Adolescent athletes’ perceived SR has been associated with a number of outcomes, including greater perceived strain (negative symptoms associated with stress) and lesser hedonic well-being (i.e., life satisfaction; Britton et al., 2017). Self-report measures of perceived SR (such as the PSRS-AA; Britton et al., 2017) provide a less costly, less invasive, and more ecologically valid alternative to lab-based measures of SR, such as cortisol sampling. Moreover, assessing SR physiologically during athletic performance runs the risk of being unable to distinguish between responses to physical demands of competition and psychological demands (Polman et al., 2010).

The perceived influence of SR on the performance of adolescent athletes, via the process of appraisal and coping, is currently not known (Britton et al., 2017). Further research is also required to examine the relationship between appraisals, emotions, coping, and performance satisfaction among adolescents, by replicating the path analysis performed by Nicholls et al. (2012). This is significant given that adolescents are known to appraise and cope with stressors differently to adults (Compas et al., 2001).

## The Present Study

**FIGURE 2:**
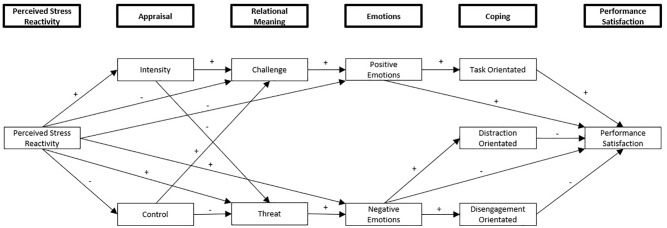
Initial hypothesized model for the relationships between PSR, competition appraisals, relational meanings, emotions, coping, and performance satisfaction.

The purpose of this study was to extend Nicholls et al.’s (2012) path analysis in two key ways: (1) To examine the direct and indirect effects of Perceived Stress Reactivity (PSR) as a dispositional variable on the stress, emotion, and coping process. (2) To examine the relationships between competition appraisals, emotions, coping and performance satisfaction within a sample of exclusively adolescent athletes, rather than adults. The hypothesized model is illustrated in Figure 2, with PSR the main predictor of the model. Arrows indicate a direct effect, plus signs infer a positive relationship, and minus signs a negative relationship.

A number of hypotheses were made regarding the different variables within the model: (1) PSR would have a direct effect on competition appraisal. In addition, it was predicted that PSR would positively predict stressor intensity (primary appraisal), and negatively predict perceived control (secondary appraisal). This was due to previous research associating adolescent athletes’ PSR with personality traits associated with greater stressor intensity and perceived lower control (Kaiseler et al., 2012a; Britton et al., 2017).

(2) Perceived Stress Reactivity would have both direct and indirect effects (via competition appraisals) on relational meaning. Specifically, PSR would positively predict perceived threat, and negatively predict perceived challenge. This is because PSR has been associated with increased threat appraisals in previous research (Schlotz et al., 2011a). It was also predicted that participants would make threat appraisals when they appraised themselves as having little perceived control, and challenge appraisals when appraising high perceived control, replicating Nicholls et al.’s (2012) findings.

(3) Perceived Stress Reactivity would have both direct and indirect effects (via competition appraisal and relational meaning) on emotion. It was predicted that PSR would positively predict negative emotion, and negatively predict positive emotion. This is because SR has been associated with negative emotionality in adolescents, and PSR has been associated with greater perceived strain overtime in adolescent athletes (Marceau et al., 2012; Britton et al., 2017). It was also predicted that threat appraisals would be associated with greater negative emotions, and challenge appraisals with positive emotions (Nicholls et al., 2012).

(4) Perceived Stress Reactivity would have an indirect effect on coping via competition appraisals, relational meaning, and emotion. PSR would positively affect disengagement and distraction orientated coping, and negatively affect task orientated coping. This was predicted because adolescent athletes’ PSR has been related to personality traits associated with coping, namely high levels of PSR with neuroticism, and low levels with emotional stability (Kaiseler et al., 2012a; Britton et al., 2017). It was also predicted that positive emotions would predict task-orientated coping, and negative emotions would predict both distraction and disengagement-orientated coping (Nicholls et al., 2012).

(5) Perceived Stress Reactivity would have a negative indirect effect on subjective performance via competition appraisals, relational meaning, emotion, and coping. Furthermore, it was predicted that emotion would have a direct and indirect effect (via coping) on subjective performance, with positive emotion predicting increased performance satisfaction and negative emotion decreased performance satisfaction. A direct effect of coping on subjective performance satisfaction was predicted, as both are affective variables, and likely to correlate irrespective of coping (Nicholls et al., 2012). Finally, coping would have a direct effect on subjective performance, with task-orientated coping predicting increased performance satisfaction, and both distraction and disengagement-orientated coping predicting decreased performance satisfaction.

No predictions were made regarding potential negative relationships between, for example, positive emotions and disengagement coping, or negative emotions and task-orientated coping, in order to avoid reducing the power of the model (Byrne, 2016).

## Materials and Methods

### Participants

Participants were 229 adolescent athletes (male *n* = 150; female *n* = 79; *M* age = 18.55, *SD* = 2.40) who competed at international/national (*n* = 8), regional (*n* = 11), county/academy (*n* = 85), club (*n* = 93), or school/university (*n* = 32) levels in the United Kingdom. Participants were recruited opportunistically from numerous sports clubs, academies, schools, and universities. They needed to be participating in competitive sport and between the ages of 12 and 22. The sample consisted of 167 adolescents from team sports (including rugby, football, and cricket) and 62 from individual sports (including golf, karate, and badminton). All participants received an information sheet and were asked to sign a consent form prior to the study. For all participants under the age of 16, parents or guardians were also sent an information sheet and asked to provide written consent.

### Materials and Methods

#### PSR

The PSRS-AA (Britton et al., 2017) was used to assess individual differences in PSR. The PSRS-AA consists 23 items over five subscales assessing reactivity to different domains: reactivity to social evaluation (“When I have to perform in front of other people…”), reactivity to social conflict (“When I have arguments with team-mates and coaches…”), reactivity to failure (“When I fail at something…”), reactivity to work overload (“When all my different training sessions and matches build up and become hard to manage…”), and prolonged reactivity (“When I want to relax after a hard training session or match…”). The aggregate score from these five subscales create an overall score of total reactivity. Each item is assessed using three descriptive multiple-choice options of differing levels of reactivity in response to a proposed stressful situation (e.g., When I have little time to prepare for a match: (a). I usually stay calm, (b). I usually feel uneasy, (c). I usually get quite unsettled). The answers reflecting lowest reactivity are scored zero, while the answers reflecting highest reactivity are scored with two. Intermediate answers are scored one. Subscales scores are calculated via the mean, with each mean subscale score being summed to calculate the aggregate measure of total reactivity. Britton et al. (2017) confirmed the hierarchal structure of the adapted scale using a second order model. The PSRS-AA’s subscales demonstrate only marginal reliability (α = 0.62 –0.73). However, the overall aggregate score of total reactivity had good reliability (α = 0.87).

#### Competition Appraisals and Relational Meanings

A version of the “stress thermometer” was used to assess primary appraisal in the form of perceived stressor intensity prior to competition (Kowalski and Crocker, 2001), with a 10 cm visual analog scale (VAS) measuring from 0 (not at all stressful) to (extremely stressful) 100. The stress thermometer has previously demonstrated normal distribution within a sample of adolescent athletes and has been utilized in many studies measuring athletes’ stressor appraisals (Kowalski and Crocker, 2001; Kaiseler et al., 2012a). In order to maintain similarity with the measure of primary appraisal, a 10-cm VAS was also used to measure secondary appraisal in the form of perceived overall control prior to competition (Kaiseler et al., 2012a), measuring from 0 (no control) to 100 (total control). To maintain further similarity and consistency with the measure of primary and secondary appraisal, levels of both challenge and threat experienced prior to competition were also measured with separate VASs, measuring from 0 (not at all a threat; not at all a challenge) to 100 (very much a threat; very much a challenge). Nicholls et al.’s (2012) original path analysis utilized the 28 item Stress Appraisal Measure (Peacock and Wong, 1990). However, it was decided that a briefer method of assessing appraisals was more suitable for the current study, in order not to burden adolescents with copious items prior to competing, especially given the addition of the 23 item PSRS-AA within this battery of tests, and thus to allow for the completion of the assessments as close to the beginning of competition as possible. The use of VAS are increasingly adopted in order to assess athletes’ appraisals of stressors and relational meaning (Turner et al., 2012; Kaiseler et al., 2012a,b; Turner et al., 2014).

#### Emotions

The Sport Emotion Questionnaire (SEQ; Jones et al., 2005) was used to retrospectively assess the emotions experienced during competition. The SEQ assesses five emotions grouped into two higher order dimensions: positive emotions (excitement and happiness) and negative emotions (anxiety, dejection, and anger). The scale contains 22 items scored on a 5-point Likert scale from 0 = “not at all” to 4 = “extremely.” The SEQ has been reported to have excellent reliability for its scales, with Cronbach’s alpha ranging from 0.81 to 0.90 (Jones et al., 2005).

#### Coping

The Coping Inventory for Competitive Sport (CICS; Gaudreau and Blondin, 2002) was used to retrospectively assess how participants coped during competition. The CICS measures ten coping subscales grouped into three coping dimensions: task-orientated coping (thought control, mental imagery, relaxation, effort expenditure, logical analysis, and support seeking), distraction-orientated coping (distancing and mental distraction), and disengagement-coping (disengagement and venting). Nine of the subscales feature four items, while one features three items. The scale uses a 5-point Likert scale to assess the extent to which the coping strategy described corresponds with what the athlete did during competition, ranging from 1 = “does not correspond at all” to 5 = “corresponds very strongly.” The CICS’s measure of three coping dimensions feature adequate to good levels of reliability (α = 0.73 to 0.87) and has been utilized with adolescent athlete populations (Nicholls et al., 2009).

The transactional model of stress and coping typically refers to three different dimensions of coping: Problem-focussed (coping designed to eliminate a source of stress), emotion-focussed (coping which addresses the emotional distress caused by a stressor), and avoidance (physically or mentally withdrawing from a stressor, Lazarus and Folkman, 1987). However, this study chose to use Gaudreau and Blondin’s (2002) task, distraction, and disengagement-orientated dimensions instead for two reasons. Firstly, this was the measure used by Nicholls et al. (2012) which this study aimed to extend. Secondly, the CICS assesses coping within the specific context of sports performance, rather than measures of Lazarus and Folkman’s dimensions, which refer to coping in much broader contexts. Therefore, it was concluded that the CICS was the most appropriate measure for assessing coping within this context.

#### Performance Satisfaction

Participants subjectively rated how satisfied they were with their performance on a VAS ranging from 0 (“not at all satisfied”) to 100 (“totally satisfied”; Pensgaard and Duda, 2003). This subjective measure of performance was used instead of an objective measure in order to compare performance across a range of different sports and positions within sports (Terry, 1995; Males and Kerr, 1996). Furthermore, subjective satisfaction provides a more sensitive measure of performance, as it can be compared between participants despite differences in environmental factors such as playing conditions, weather, or opponents’ skill levels (Nicholls et al., 2012).

### Procedure

University ethics board approval was obtained prior to data collection. Participants firstly completed the PSRS-AA prior to competition. The VAS measures of competition appraisals and relational meaning were then completed less than 1 h before competing at a time and place agreed with by the researcher, participant, and coach if one was present. The SEQ, CICS and VAS measure of performance satisfaction was completed less than 1 h after competing also at an agreed time.

### Data Analysis

The proposed path analysis containing PSR, competition appraisals, relational meanings, emotions, coping, and performance satisfaction was tested in SPSS Amos (v.24) using maximum likelihood estimation. This allows for the simultaneous examination of direct and indirect effect paths throughout the model, while also testing the overall fit of the data to the hypothesized model (Byrne, 2016). For structural equation models, 200 cases are considered a minimum requirement as a rule of thumb (Kline, 1998). The following variables were originally entered: PSR, stressor intensity, perceived control, threat, challenge, negative emotions, positive emotions, task-orientated coping, distraction-orientated coping, disengagement-orientated coping, and performance satisfaction (see Figure 2). The error terms of distraction and disengagement-orientated coping were allowed to co-vary with one another, as they were anticipated to correlate. No other co-variances between shared antecedents were drawn, as no more correlations were predicted based on existing theory. Bivariate correlations were calculated in order to initially analyze the relationships between the variables entered into the model.

A number of indices were used to assess overall model fit. The chi-square statistic assesses the magnitude of discrepancy between the data sample and the co-variance matrix predicted by the model (Hu and Bentler, 1999). However, chi-square is notably sensitive to sample size. Therefore, the chi-square/degrees of freedom ratio (CMIN/DF) was used in order to minimize the effect of sample size on determining model fit (Hooper et al., 2008). A threshold of 3 was used to indicate an acceptable model fit (Kline, 1998). The comparative fit index (CFI) was assessed in order to indicate the extent to which the theoretical model better fitted the data in comparison to a base model where all constructs are constrained to be correlated with one another, with greater than or equal to 0.95 indicating good model fit, and 0.90 indicating adequate fit (Hu and Bentler, 1999; Hooper et al., 2008). Root mean square error of approximation (RMSEA) was calculated in order to provide an estimate of the average absolute difference between estimated model covariances and the observed covariances, with less than 0.06 indicating good model fit (Hu and Bentler, 1999; Hooper et al., 2008). A *p*-value testing the null hypothesis (PCLOSE) of the RMSEA was also assessed, with a non-significant result greater than 0.05 required to reject the null.

Standardized regression (beta) weights were used to examine the size and significance of the direct effects of PSR specified within the model (Byrne, 2016). To examine the indirect effects of PSR through the model, the probability associated with the standardized indirect effects and their respective confidence intervals (90%) were estimated using a bias-corrected confidence interval bootstrap test (using 500 samples; Byrne, 2016).

### Data Preparation

Prior to conducting the path analysis, data were screened for outliers and normality. Univariate normality was assessed using skewness and kurtosis values, while multivariate normality was examined using Malhalanobis distances. Seven cases were removed from the analyses due to the presence of multivariate outliers. To test the validity of the questionnaire measures used, confirmatory factor analyses (CFA) using SPSS Amos (v.24) were performed on the SEQ and the CICS. This was to test the fit of the scales and subscales to their proposed higher order structures, so modifications (such as item co-variances or removals) could be made to the scales if required. This would confirm validity of the scale for use with the sample population. The same goodness of fit indices were used.

The positive emotion dimension of the SEQ provided good model fit once two co-variances were drawn between the error terms of items 5 and 10, and items 10 and 20 on the happiness subscale (CMIN/DF = 1.73; CFI = 0.99; RMSEA = 0.06; PCLOSE = 0.34). The negative emotion dimension provided good model fit once two co-variances were drawn between the error terms of items 2 and 7 on the dejection subscale and 9 and 19 on the anger subscale, and item 1 was removed from the anxiety subscale due to multiple high modification indices with items on other subscales (CMIN/DF = 1.95; CFI = 0.98; RMSEA = 0.06; PCLOSE = 0.11). The combined model for the whole questionnaire, however, produced questionable model fit (CMIN/DF = 1.98; CFI = 0.95; RMSEA = 0.07; PCLOSE = 0.01). This may have been due to large covariances between the anxiety subscale and happiness subscale from the positive dimension. Mean scores for the subscales and dimensions of the SEQ were then calculated based upon these modifications.

The task-orientated dimension of the CICS provided adequate model fit after co-variances were drawn between the error terms of items 18 and 28 on the relaxation subscale, and items 9 and 29 on the logical analysis subscale (CMIN/DF = 1.73; CFI = 0.91; RMSEA = 0.06; PCLOSE = 0.12). The distraction subscale provided good model fit once item 3 was removed from the social withdrawal subscale due to large co-variances with items on the mental distraction subscale (CMIN/DF = 1.79; CFI = 0.96; RMSEA = 0.06; PCLOSE = 0.31). The disengagement subscale provided adequate model fit once items 22 and 32 were removed from the venting subscale due to large co-variances with the disengagement subscale (CMIN/DF = 2.99; CFI = 0.97; RMSEA = 0.09; PCLOSE = 0.04). However, no further modifications were made, as CFI indicated good model fit. The three dimensions combined into one model also provided questionable model fit, with no indications that further modifications would improve the model (CMIN/DF = 1.85; CFI = 0.84; RMSEA = 0.06; PCLOSE = 0.00). However, given that the individual dimensions provided good to adequate model fits, analysis proceeded. Mean scores for the subscales and dimensions of the CICS were then calculated based upon these modifications.

## Results

Table 1 provides means, standard deviations, and Cronbach’s alpha coefficients for all the variables entered in the model, including discrete emotions and coping strategies. Table 2 provides Pearson’s *r* correlations between all variables entered into the model. Table 3 provides correlations between the discrete coping strategies measured by the CICS and performance satisfaction.

**Table 1:** Mean and standard deviations for variables used in model and Cronbach’s alpha coefficients.

Scales	Mean	SD	α
Prolonged Reactivity	0.42	0.36	0.48
Reactivity to Work Overload	0.45	0.38	0.57
Reactivity to Social Conflict	0.62	0.40	0.68
Reactivity to Failure	0.93	0.40	0.68
Reactivity to Social Evaluation	0.57	0.42	0.66
Total Reactivity	3.01	1.45	0.85
Intensity	42.25	23.63	
Control	61.57	23.52	
Challenge	61.46	20.96	
Threat	35.27	22.70	
Excitement	2.61	0.91	0.81
Happiness	2.63	1.09	0.89
Positive emotions	2.62	0.92	0.90
Anxiety	1.53	0.97	0.89
Dejection	1.15	0.88	0.88
Anger	1.58	0.94	0.87
Negative emotions	1.42	0.77	0.90
Thought control	2.95	0.87	0.68
Mental imagery	2.75	0.84	0.68
Relaxation	2.33	0.98	0.84
Effort	3.96	0.72	0.75
Logical analysis	2.76	0.84	0.68
Seeking support	2.21	0.91	0.76
Task-orientated coping	2.83	0.61	0.89
Social withdrawal	1.79	0.70	0.55
Mental distraction	1.60	0.62	0.67
Distraction orientated coping	1.70	0.57	0.73
Venting	2.47	1.21	0.72
Disengagement	1.44	0.60	0.76
Disengagement orientated coping	1.96	0.73	0.68
Performance satisfaction	63.90	22.56	

**Table 2:** Pearson’s *r* correlations between all variables entered into the model.

Scales	1	2	3	4	5	6	7	8	9	10
1. Total reactivity										
2. Intensity	0.34^∗∗^									
3. Control	−0.23^∗∗^	−0.15^∗^								
4. Challenge	0.15^∗^	0.52^∗∗^	−0.04							
5. Threat	0.29^∗∗^	0.54^∗∗^	−0.07	0.47^∗∗^						
6. Positive emotions	0.10	−0.02	0.10	0.02	−0.05					
7. Negative emotions	0.21^∗∗^	0.24^∗∗^	−0.21^∗∗^	0.27^∗∗^	0.21^∗∗^	−0.04				
8. Task orientated coping	−0.04	−0.12	0.25^∗∗^	0.05	0.01	0.42^∗∗^	0.06			
9. Distraction orientated coping	0.08	0.00	0.07	0.07	0.04	0.08	0.18^∗∗^	0.48^∗∗^		
10. Disengagement orientated coping	0.26^∗∗^	0.14^∗^	0.00	0.09	0.15^∗^	−0.02	0.40^∗∗^	0.15^∗^	0.29^∗∗^	
11. Performance satisfaction	−0.06	0.12	0.22^∗∗^	−0.07	−0.10	0.52^∗∗^	−0.36^∗∗^	0.15^∗^	−0.16^∗^	−0.29^∗∗^

*Note. ^∗^p < 0.05., ^∗∗^ p < 0.01.*

**Table 3:** Pearson’s *r* correlations between discrete coping strategies and performance satisfaction.

Scales	1	2	3	4	5	6	7	8	9	10
1. Thought control										
2. Relaxation	0.49^∗∗^									
3. Effort	0.33^∗∗^	0.19^∗∗^								
4. Logical analysis	0.52^∗∗^	0.59^∗∗^	0.33^∗∗^							
5. Mental imagery	0.51^∗∗^	0.50^∗∗^	0.39^∗∗^	0.66^∗∗^						
6. Seeking support	0.25^∗∗^	0.36^∗∗^	0.20^∗∗^	0.39^∗∗^	0.38^∗∗^					
7. Mental distraction	0.26^∗∗^	0.23^∗∗^	0.03	0.20^∗∗^	0.30^∗∗^	0.27^∗∗^				
8. Social withdrawal	0.41^∗∗^	0.48^∗∗^	0.07	0.43^∗∗^	0.36^∗∗^	0.31^∗∗^	0.49^∗∗^			
9. Venting	0.24^∗∗^	0.01	0.01	0.19^∗∗^	0.15^∗^	0.06	0.11	0.18^∗∗^		
10. Disengagement	0.07	0.10	−0.24^∗∗^	0.05	0.00	0.16^∗^	0.31^∗∗^	0.30^∗∗^	0.23^∗∗^	
11. Performance satisfaction	0.07	0.02	0.32^∗∗^	0.10	0.20^∗∗^	0.01	−0.10	−0.17^∗^	−0.17^∗^	−0.38^∗∗^

*Note. ^∗^p < 0.05, ^∗∗^p < 0.01.*

To examine the overall fit of all the data collected, the model shown in Figure 2 was tested. The fit of the model produced inadequate model fit (CMIN/DF = 4.29; CFI = 0.79; RMSEA = 0.12; PCLOSE < 0.01). Based upon modification indices and correlations within the data set, modifications were made to the model in the form of additional paths. These modifications were only made if they were theoretically sound and did not fundamentally change the nature of the path (Nicholls et al., 2012). An additional path was drawn from control to both negative emotions, and from control to task-orientated coping, as both demonstrated high modification indices, and existing theory would suggest that secondary appraisal of control and coping resources has the potential to directly influence the experience of negative emotions and the use of adaptive coping strategies ( Lazarus and Folkman, 1987; Lazarus, 1999; Fletcher et al., 2006).

The overall revised model, however, still produced inadequate fit (CMIN/DF = 3.96; CFI = 0.82; RMSEA = 0.12; PCLOSE < 0.01). Figure 3.1, 3.2 both illustrate the final model, with separate figures for the “positive” and “negative” paths used for ease of illustration. The significance levels of each path coefficient are included. Table 4 details the direct and indirect effects (plus bias corrected confidence intervals) for PSR and all other variables included in the final model.

**FIGURE 3:**
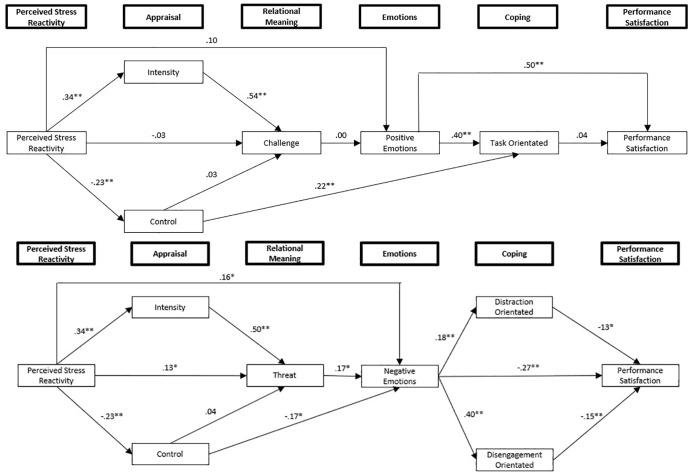
Revised model of relationships between PSR, competition appraisals, challenge, positive emotions, task-orientated coping, and performance satisfaction. Revised model of relationships between PSR, competition appraisals, threat, negative emotions, distraction and disengagement orientated coping, and performance satisfaction.

**Table 4:** Direct and indirect effects of variables entered into the model.

			Indirect	
Independent Variable	Dependent Variables	Direct	Sum	90% CI
PSR	Intensity	0.34^∗∗^		
	Control	−0.23^∗∗^		
	Challenge	−0.03	0.18^∗∗^	0.11.25
	Threat	0.13^∗^	0.16^∗∗^	0.10,0.23
	Positive Emotions	0.10	0.00	−0.02,0.02
	Negative Emotions	0.16^∗^	0.09^∗∗^	0.04,0.15
	Task-orientated		−0.01	−0.07,0.05
	Distraction-orientated		0.04^∗∗^	0.01,0.08
	Disengagement-orientated		0.08^∗∗^	0.03,0.12
	Performance satisfaction		−0.02	−0.09,0.05
Intensity	Challenge	0.54^∗∗^		
	Threat	0.50^∗∗^		
	Positive Emotions		0.00	−0.06,0.06
	Negative Emotions		0.08^∗^	0.02,0.16
	Task-orientated		0.00	−0.03,0.02
	Distraction-orientated		0.01^∗^	0.00,0.03
	Disengagement-orientated		0.03^∗^	0.01,0.07
	Performance satisfaction		−0.03	−0.07,0.01
Control	Challenge	0.03		
	Threat	0.04		
	Positive Emotions		0.00	−0.00,0.00
	Negative Emotions	−0.17^∗^	0.01	−0.01,0.02
	Task-orientated	0.22^∗∗^	−0.00	−0.00,0.00
	Distraction-orientated		0.04^∗^	0.01,0.08
	Disengagement-orientated		0.08^∗^	0.03,0.12
	Performance satisfaction		−0.02^∗^	−0.09,0.05
Challenge	Positive Emotions	0.00		
	Task-orientated		0.00	−0.04,0.05
	Performance satisfaction		0.00	−0.06,0.06
Threat	Negative Emotions	0.17^∗^		
	Distraction-orientated		0.03^∗^	0.01,0.06
	Disengagement-orientated		0.07^∗^	0.02,0.12
	Performance satisfaction		−0.06^∗^	−0.11, −0.01
Positive Emotions	Task-orientated	0.40^∗∗^		
	Performance satisfaction	0.50^∗∗^	0.02	−0.02,0.06
Negative Emotions	Distraction-orientated	0.18^∗∗^		
	Disengagement-orientated	0.40^∗∗^		
	Performance satisfaction	−0.27^∗∗^	−0.08^∗∗^	−0.14, −0.03
Task-orientated	Performance satisfaction	0.04		
Distraction-orientated	Performance satisfaction	−0.13^∗^		
Disengagement-orientated	Performance satisfaction	−0.15^∗∗^		

*Note. ^∗^p < 0.05, ^∗∗^p < 0.01.*

## Discussion

In this study, a path analysis was used to examine adolescent athletes’ PSR, competition appraisals and relational meanings prior to competition, emotions and coping strategies during competition, and subjective performance satisfaction. This was to explore the direct and indirect effects of PSR on the stress and coping process of adolescent athletes, plus to further extend the path analysis conducted by Nicholls et al. (2012). The revised models (Figure 3.1, 3.2) did not provide adequate model fit, which limits the overall conclusions which can be drawn from the model. However, there were several significant direct and indirect effects observed within the model relating to the a-priory predictions (see Table 4). These will be discussed in turn. The study extends the understanding of how perceived SR influences the stress, emotion, and coping process among adolescent athletes. The study also provides a further examination of stress appraisal and coping measures, and their validity among adolescent athlete populations.

Perceived Stress Reactivity demonstrated direct effects on competition appraisals of intensity and control, relational meanings of threat, and negative emotions. PSR also demonstrated indirect effects on threat, challenge, negative emotions, and maladaptive coping (distraction and disengagement-orientated coping). However, PSR failed to demonstrate effects (direct or indirect) on positive emotions, task-orientated coping, or performance satisfaction. Although the analyses shared some similarities with Nicholls et al. (2012), there were also a number of divergences. Overall, these findings provide new information on how PSR influences the stress and coping process, as well as how competition appraisals, emotions, and coping impact upon the performance satisfaction of adolescent athletes (see Table 4). In addition, findings suggest there are some differences in the stress and coping process in adolescents compared to adult athletes.

In relation to the first set of hypotheses, participants with higher levels of PSR were more likely to appraise the impending competition as more stressful, and to appraise themselves as having less control, and thus not have the resources to cope. This is consistent with previous research which has found individual differences (most notably neuroticism) to predict athletes’ appraisals of stressor intensity and perceived control (Kaiseler et al., 2012a). These are among the strongest effects within the model, confirming that an adolescent athletes’ perception of how reactive they are to stressors in general has a direct effect on how they cognitively appraise sporting competitions.

With regards to the second set of hypotheses, adolescent athletes with a higher level of PSR were more likely to form a relational meaning of threat in relation to the impending competition. This was partially due to their increased likelihood of scoring the stress relating to the impending competition as more intense. This is consistent with previous research which has associated measures of PSR with increased threat appraisals (Schlotz et al., 2011a). However, participants with higher levels of PSR were also more likely to appraise the impending competition as a challenge, via the increased appraisal of intensity. This suggests that appraisal is not dichotomous, and that athletic competition can be appraised with a level of challenge and threat at the same time. This supports previous research that has suggested that challenge and threat appraisals can co-occur (Lazarus, 1999). In this sample, it is possible that adolescent athletes with high levels of PSR were more likely to appraise competitions with greater personal relevance (primary appraisal), hence greater appraisals of both challenge and threat.

Control appraisals, however, did not influence the relational meaning of either threat or challenge. Such a finding does not support theory and previous empirical findings with adult populations that has associated secondary stressor appraisals with relational meanings of challenge and threat (Lazarus and Folkman, 1987; Fletcher et al., 2006; Nicholls et al., 2012). This might suggest that, for adolescent athletes, factors outside of their own perceived personal control of the situation, may account for the secondary appraisals that determine relational meanings of challenge or threat (e.g., perceived social support from others). In Nicholls et al.’s (2012) original model, the full 28 item SAM was used, which assesses not only controllability by the self, but also the perceived controllability of stressors by others. By using only a single item in this study, this may not have sufficiently captured the full nature of secondary appraisal, and how an adolescent athlete perceives the resources available to them, thus determining whether situations of high personal relevance and stressfulness are perceived as either challenges or threats.

Adolescent athletes who viewed themselves as having greater control prior to competition did experience fewer negative emotions and used more task-focussed coping strategies. This is consistent with previous empirical findings and theory, suggesting that if adolescent athletes were to perceive themselves as having a high level of control the impending competition, they would have significant resources available to cope and thus would likely experience less negative emotions and have a larger repertoire of task-focussed coping strategies (Lazarus and Folkman, 1987; Amiot et al., 2004; Fletcher et al., 2006; Neil et al., 2011; Nicholls et al., 2012).

In relation to the third set of hypotheses, adolescent athletes with higher levels of PSR were more likely to experience negative emotions during competition. This is explained directly by an adolescent athletes PSR, and indirectly via cognitive appraisal. This supports previous research that has associated increased reactivity in adolescents with negative emotionality (Marceau et al., 2012). PSR, however, did not feature any direct or indirect effects on positive emotions. Like appraisal, negative and positive emotions can co-exist (Folkman, 1997; Polman and Borkoles, 2011). Adolescent athletes’ experience of positive emotions is likely to be determined by other factors which we did not measure in the current study. With regards to appraisals predicting emotions, supporting previous findings, threat was positively associated with negative emotions (Lazarus, 2000; Nicholls et al., 2012). Similarly, decreased control also predicted negative emotions. However, challenge did not predict positive emotions as expected. As indicated previously, the sample characteristics (adolescent athletes) and the way appraisal was measured in the present study might explain this finding. The notion that positive emotions experienced by adolescent athletes are not predicted by any antecedents within the present study supports findings that the stress-coping process in adolescents is different compared to that of adults (Davis and Compas, 1986; Compas et al., 2001).

With regards to the fourth set of hypotheses, adolescent athletes with high levels of PSR were more likely to use coping strategies during competition that are considered maladaptive, via increased threat appraisals and negative emotions. This supports previous research which has observed an association between athletes’ individual differences and maladaptive coping (e.g., Kaiseler et al., 2012a). However, no effects were observed between PSR and task-orientated coping. These findings point toward the notion that the PSR is more likely to predict the maladaptive aspects of high SR (more negative emotions, maladaptive coping) but that less SR is not automatically associated with adaptive outcomes (positive emotions, adaptive coping). However, although no relationship was found between PSR and adaptive coping, it is possible that low levels of PSR may be facilitative via other processes not observed within the model (e.g., via lower levels of negative emotion). Supporting previous findings (Nicholls et al., 2012; Laborde et al., 2016), positive emotions predicted the use of task-orientated coping, and negative emotions predicted both distraction and disengagement-orientated coping.

In relation to the fifth and final set of hypotheses, PSR was found to have no indirect effect on subjective performance satisfaction via the stress and coping process experienced prior to and during competition. This suggests that, in the short-term, high levels of PSR do not have an impact upon the subjective performance of adolescent athletes on the day of competition. However, this is not to say that PSR does not impact upon adolescent athletes’ actual and subjective performance and well-being in the long-term. Youth athletes’ PSR is associated with increased strain over a 30 day period and decreased life-satisfaction (Britton et al., 2017). Furthermore, athletes experience multiple organizational stressors, other than those in competition, which can impact upon performance (Mellalieu et al., 2009; Arnold et al., 2017). Therefore, PSR may influence the appraisal of other organizational stressors experienced by adolescent athletes (such as conflicts with team-mates or training) which may in turn impact upon emotions, coping, and performance in the long-term.

Similar to Nicholls et al. (2012), positive emotions in the adolescent athletes were directly associated with higher and negative emotions with lower levels of subjective performance satisfaction. This association is not unexpected because both are affective variables. At the dimensional level the use of distraction and disengagement coping significantly predicted lower levels of subjective performance satisfaction as expected. However, task-oriented coping was not directly associated with higher levels of performance satisfaction. The correlation matrix showed that the only task-oriented coping strategies associated with subjective performance were mental imagery and effort (see Table 3). This suggests that the majority of the task-orientated coping strategies proposed by Gaudreau and Blondin (2002) are not associated with increased performance. This is not consistent with adult samples which have found a much wider range of coping strategies from the CICS to predict performance satisfaction (see Nicholls et al., 2012). This suggests that adolescent athletes have a much smaller range of effective coping strategies compared to adults. The effectiveness of these strategies, and thus the effect on performance satisfaction, may be explained by maturational processes, as adolescent athletes’ coping effectiveness has been shown to increase with emotional and social maturity (Nicholls et al., 2013, 2015).

Similar to Nicholls et al. (2012), negative emotions indirectly predicted subjective performance satisfaction via distraction and disengagement-oriented coping. However, there was no indirect effect for positive emotions. Overall, the direct and indirect effects on subjective performance satisfaction suggest that adolescent athletes’ emotions experienced during competition are greater predictors of performance satisfaction than the coping strategies they use. Specifically, although maladaptive coping strategies predict decreased performance satisfaction, the task-orientated strategies considered effective by adults (Gaudreau and Blondin, 2002; Nicholls et al., 2012) are not associated with increased performance satisfaction among adolescents.

### Practical Implications

For applied practitioners, these findings have a number of implications. Firstly, practitioners can use the PSRS-AA to identify adolescent athletes most likely to appraise competitions with greater intensity, less perceived control, greater perceived threat, more likely to experience negative emotions, and more likely to use maladaptive coping strategies. Having identified adolescents at greatest risk, practitioners could employ a range of interventions to help athletes manage the effects of reactivity on stress and its outcomes. Given that stress is a recursive process (Lazarus, 1999) and that reactivity is a variable disposition (Schlotz et al., 2011b), successful interventions could bring about long-term adaptations in reactivity over time.

Specifically, given the findings within this sample, adolescent athletes with high levels of PSR could be prioritized for interventions that address control appraisals prior to competitive performances. Although control appraisals were not related to relational meanings of challenge or threat within this sample, they were associated with fewer negative emotions during competition and the greater use of task-orientated coping strategies. Manipulating athletes’ appraisals of their resources available to them has been found to positively impact upon physiological responses to pressure situations (Turner et al., 2014).

Given the recursive nature of stress (Lazarus, 1999), coping interventions could also prove effective in assisting adolescent athletes with high level of PSR. Enhancing and refining an adolescent’s coping repertoire is likely to affect future control appraisals, by increasing coping self-efficacy (Reeves et al., 2011). Although previous research has recommended that athletes use a wide range of task-orientated strategies to enhance performance (Gaudreau and Blondin, 2002; Nicholls et al., 2012), correlations within the present data set would suggest that effort expenditure and mental imagery could be taught as coping strategies to adolescent athletes to enhance their performance (see Table 3).

Finally, given the direct and indirect effects of PSR on the negative emotions, interventions based upon the processes of emotion regulation could also be recommended for young athletes measuring highly on the PSRS-AA (Lane et al., 2012; Wagstaff, 2014).

### Study Strengths

This study has several strengths and provides a number of novel findings. Few studies have examined the associations between competition appraisals, emotions, coping, and performance satisfaction using longitudinal data, let alone with adolescents. The focus on adolescents in this study extends the work Nicholls et al. (2012) with adult athletes. Furthermore, this study also extends existing research by examining the direct and indirect effects of a dispositional factor (PSR) on the stress and coping process. Specifically, the strong associations between PSR and competition appraisals (perceived intensity and control) enhance the validity of the PSRS-AA as a measure of adolescent athletes’ individual differences in reactivity, capable of predicting psychological responses to competition stressors.

### Study Limitations

A general weakness of the study can be found within the reliance on self-report measures, which are associated with numerous biases (Furnham, 1986). Furthermore, there appear to be specific limitations with the single item VAS measures of appraisal and relational meaning utilized within the study. The measures of relational meaning (challenge and threat) were significantly positively correlated and were not associated with secondary appraisals of control. This brings into question the validity and reliability of the use of single item VAS scales to measure stress appraisal, despite their use in previous research (Kaiseler et al., 2012a,b; Turner et al., 2012, 2014). In other words, single item VAS scales may not be sufficient for capturing the complex nature of stress appraisal adolescent athletes undergo prior to competition.

The SAM (Peacock and Wong, 1990) used by Nicholls et al. (2012) may have been a more comprehensive measure of appraisal and relational meaning, despite the burden its length may have placed upon participants required to complete it close to the start of competition. Alternatively, given that athletes experience multiple stressors prior to competition other than just the competition itself (Mellalieu et al., 2009), assessing just the appraisals and relational meanings of the competition may have been too broad for capturing the dynamic nature of stressors experienced.

The measures of task-orientated coping and distraction-orientated coping were also correlated within the sample. This is a relationship not previously observed between these two variables in both adult or adolescent samples, given that task-orientated strategies are considered adaptive, while distraction-orientated strategies are considered maladaptive (Gaudreau and Blondin, 2002; Nicholls et al., 2009; Nicholls et al., 2012). Given the dynamic nature of sporting competition, athletes, have been known to use coping strategies from across dimensions (Nicholls et al., 2007; Nicholls and Polman, 2007). Only effort and mental imagery from the task-orientated dimension correlated with performance satisfaction. However, coping strategies perceived as effective are not always associated with performance satisfaction (Didymus and Fletcher, 2017). Therefore, future research may wish to further explore the validity of the CICS for use with adolescent athletes or use alternative measures of coping validated for use with adolescent athletes (Kowalski and Crocker, 2001).

The positive and negative dimensions of the SEQ when combined also produced poor model fit. Co-variances were observed between items on the anxiety and happiness subscales, suggesting that both positive and negative emotions co-occurred within the sample, rather than being experienced distinctly. This would imply that, within adolescent athletes, SEQ may not be able to successfully distinguish between discrete positive and negative emotions as expected.

Finally, a number of potentially significant negative relationships were not specified within the model, in order to avoid reducing the overall power of the model. Low levels of negative emotion may have facilitated task orientated coping, for example, or threat appraisals associated with low levels of positive emotion. These negative associations would be expected theoretically (Lazarus and Folkman, 1987; Lazarus, 1999). However, adding too many co-variances within such a path analysis is likely to risk reducing the overall power of the model (Byrne, 2016).

### Future Research

Future research may wish to examine the factors that contribute to the development of SR in adolescent athletes. With a growing understanding of the outcomes associated with PSR in adolescent athletes (Britton et al., 2017), and how its influences the stress and coping process, youth sport organizations may benefit from an understanding of the developmental factors which contribute to some adolescent athletes having higher levels of reactivity than others. Exposure to stressors and support during childhood have already been associated with the development of reactivity in the wider population (Boyce and Ellis, 2005; Hughes et al., 2017). Future research could examine the relationship between adolescent athletes’ history of stressors and support experienced within youth support environments and their PSR using the PSRS-AA.

Given that PSR appears to be related almost exclusively to negative constructs within the analysis (threat, negative emotion, maladaptive coping), future research may also wish to examine further salutogenic constructs that may explain more positive outcomes (challenge appraisals, positive emotion, task-orientated coping). For example, mental toughness has already been associated with increased appraisals of control, and greater use of effective coping strategies (Kaiseler et al., 2009). Future studies may also wish to examine the relationship between SR and salutogenic constructs such as mental toughness or resilience.

## Conclusion

In conclusion, this study demonstrates the direct and indirect effects of individual differences in adolescent athletes’ PSR on competition appraisals, emotions, and coping. Furthermore, the study extends previous research by examining the relationship between competition appraisals, emotions, coping, and performance satisfaction within adolescent athletes. Overall model fit was not achieved, limiting the overall conclusions that can be made regarding the stress-coping process. However, several significant direct and indirect effects were observed within the path analysis, partially replicating previous research (Nicholls et al., 2012) and supporting the extant theory to some extent (Lazarus and Folkman, 1987; Lazarus, 1999; Fletcher et al., 2006). This has implications for applied practitioners, as the PSRS-AA could be used to identify young athletes who are at greater risk of experiencing negative emotions and employing maladaptive coping strategies. Practitioners’ resources could therefore be more efficiently allocated to adolescents at greatest risk. However, to inform future research further, researchers may wish to explore the validity of measures used to assess adolescent athletes’ appraisals (particularly challenge and threat) and use of coping strategies, due to divergent and null findings within the present data. Furthermore, future research may also wish to investigate which factors influence the development of SR in adolescent athletes.

## Ethics Statement

This study was carried out in accordance with the recommendations of Bournemouth University’s Research Ethics Code of Practice, with written informed consent from all subjects. For all subjects under the age of 16, written and informed consent was also provided by a parent or guardian. All subjects gave written informed consent in accordance with the Declaration of Helsinki. The protocol was approved by Bournemouth University’s Research Ethics Committee (ref: 12462).

## Author Contributions

DB, EK, and RP conceived and designed the study and experiments and edited, critically revised manuscript and approved the final version of manuscript. DB analyzed the data and drafted manuscript and prepared table/figures. DB and RP interpreted results of research.

## Conflict of Interest Statement

The authors declare that the research was conducted in the absence of any commercial or financial relationships that could be construed as a potential conflict of interest.
